# Case report: a cataract induced by bleomycin in a patient with testicular cancer

**DOI:** 10.3389/fphar.2024.1339662

**Published:** 2024-06-20

**Authors:** Wan Zhang, Yinghua Ji, Yufei Sun, Keya Zhi, Han Yang, Min Zhang, Ping Lu, Yana Zhang

**Affiliations:** ^1^ Department of Oncology, The First Affiliated Hospital of Xinxiang Medical University, Xinxiang, Henan, China; ^2^ Department of Life Science Research Center, The First Affiliated Hospital of Xinxiang Medical University, Xinxiang, Henan, China

**Keywords:** testicular cancer, bleomycin, cataract, adverse effects, chemotherapy

## Abstract

**Background:**

Bleomycin is a glycopeptide antibiotic with outstanding anti-tumor effects. A major adverse effect of bleomycin is lung fibrosis. However, the development of cataracts as a severe adverse effect has not been reported.

**Case summary:**

Herein, we describe the first case of cataract induced by bleomycin therapy in a 22-year-old male with testicular cancer. After surgical intervention and following five successive chemotherapy cycles of the BEP regimen, including bleomycin, etoposide and cisplatin, the patient reported a gradual painless loss of vision, with substantial decline in visual ability, especially in the right eye. Following comprehensive eye examinations, a cataract was diagnosed. Eventually, the patient underwent phacoemulsification and received replacement of the intraocular lenses.

**Conclusion:**

Bleomycin can cause cataracts, which induces a significant loss of vision. Therefore, clinicians should observe early symptoms and properly adjust treatment to prevent aggravation of symptoms.

## 1 Introduction

Testicular cancer is the most common genital system cancer in young men. Testicular cancer is a curable cancer with a 95% 5-year relative survival rate (Siegel et al., 2023). Platinum-based chemotherapy shows outstanding clinical efficacy (Chovanec and Cheng, 2022), which increases the long-term survival rates of patients with metastatic testicular cancer from 10% to approximately 70% (Einhorn, 1981). At present, the combination of bleomycin, etoposide, and cisplatin (BEP) is the standard treatment for testicular germ cell tumors (Alsdorf et al., 2019).

The BEP regimen can cause a series of adverse effects, including neutropenia, anemia, and thrombocytopenia, and even induces serious unusual gastrointestinal and skin toxicity (Borbélyová et al., 2020; Lauritsen et al., 2020). Bleomycin, which is glycopeptide antibiotic, is a vital part of the BEP regimen. It was first isolated by Umezawa et al., in 1966. It can be used to treat squamous cell carcinoma, Hodgkin’s disease, non-Hodgkin’s disease, testicular cancer, and malignant pleural effusion (Brandt and Gerriets, 2023). Bleomycin induces single-strand DNA breaks (SSB) and double-strand DNA breaks (DSB) and binds transition metals including Fe (II) or Cu (I) and oxygen (Czaja et al., 2022; Czaja et al., 2022). Bleomycin can induce several adverse effects including fever and chills, lung fibrosis, rash, vomiting, loss of appetite, and flagellate dermatitis (Imhof and Tollefson, 2019). Fever and chills are common adverse effects that always occur within 3–6 h after a dose. The main severe adverse effect of bleomycin is lung fibrosis, whose occurrence is higher in older patients. This adverse effect can affect up to 46% of the total patient population and leads to death in 3% of patients. A rare adverse effect of flagellate dermatitis has been observed in 8%–20% of treated patients (Chen and Stubbe, 2005; Diao and Goodall, 2012).

However, bleomycin can also induce an exceedingly rare adverse effect cataract which was previously described in newborn rats (Edwards et al., 1975). Here, we present the case of a young patient who developed a cataract induced by bleomycin. This patient was diagnosed with testicular cancer, and using bleomycin to anti-tumor. He had no history of eye disease and during the whole treatment process, the patient never used other drugs that can induce ocular toxicity.

## 2 Case description

The patient was a 22-year-old Asian male. In April 2022, the patient was admitted to the First Affiliated Hospital of Xinxiang Medical University (Xinxiang, Henan Province, China) with abdominal pain of 7-day duration. Physical examination showed that the left testicle was hard and palpable, with no firmness. Chest computed tomography (CT) and abdominal magnetic resonance imaging (MRI) revealed multiple abnormal masses. Specifically, a mass in the seminal vesicle area measured 25.8 mm*44.8 mm*31.7 mm. Additionally, there was an enlargement of a left lung mass measuring 8.0 mm*7.0 mm, and a left adrenal gland mass measured 41.6 mm*46.6 mm*49.4 mm. These findings suggest a high likelihood of metastasis. Based on the history and imaging examinations, the patient was diagnosed with testicular cancer in the final stage cT2N3M1a IIIC. On 22 April 2022, the patient received orchiectomy and epididymoidectomy. Biopsy pathological results suggested embryonal carcinoma (Supplementary Figures S1, S2). The postoperative pathological stage was pT2N3M1a IIIC stage.

**FIGURE 1:**
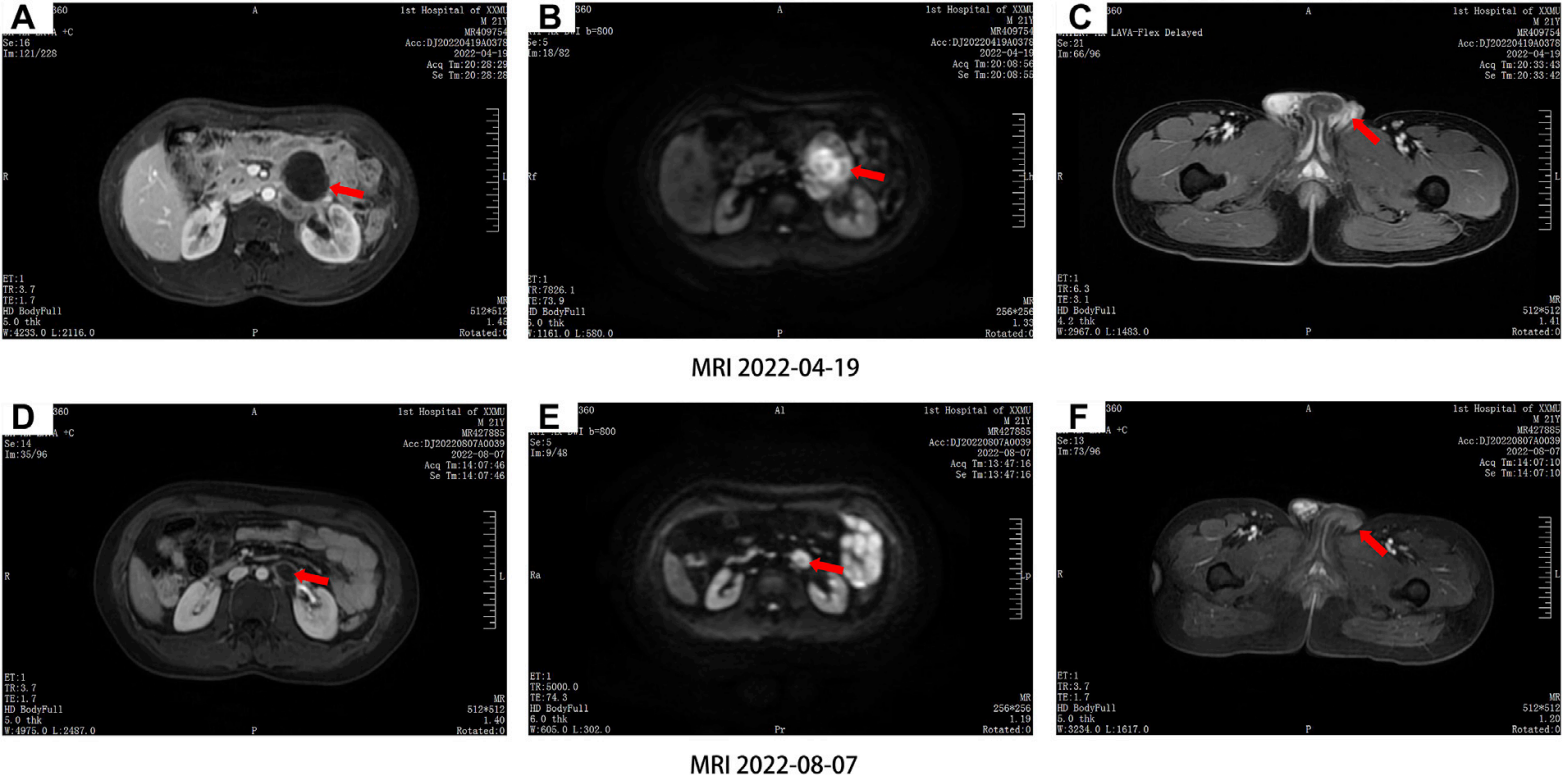
Comparison of abdominal magnetic resonance imaging **(A,B,C)** before and **(D,E,F)** after chemotherapy, the left retroperitoneal lymph nodes were significantly reduced.

After the operation, the patient received the BEP chemotherapy via intravenous infusion, which consisting of 13,000 IU bleomycin on days 1–3, with the addition of 100 mg etoposide on days 1–3, and 100 mg cisplatin on day 1 at 21-day cycle intervals. From 6 May 2022 to 16 July 2022, the patient received four successive cycles of BEP chemotherapy regimen. During BEP treatment, the patient experienced grade II myelosuppression, elevated aminotransferase levels and decreased appetite. After symptomatic treatment, these adverse effects were decreased. After the four cycles, the left adrenal lesion had reduced by 53.6% (18.1 mm*12.7 mm*22.9 mm) (Figure 1). To further enhance antitumor activity, the patient received the fifth cycle of BEP chemotherapy after careful consideration.

**FIGURE 2:**
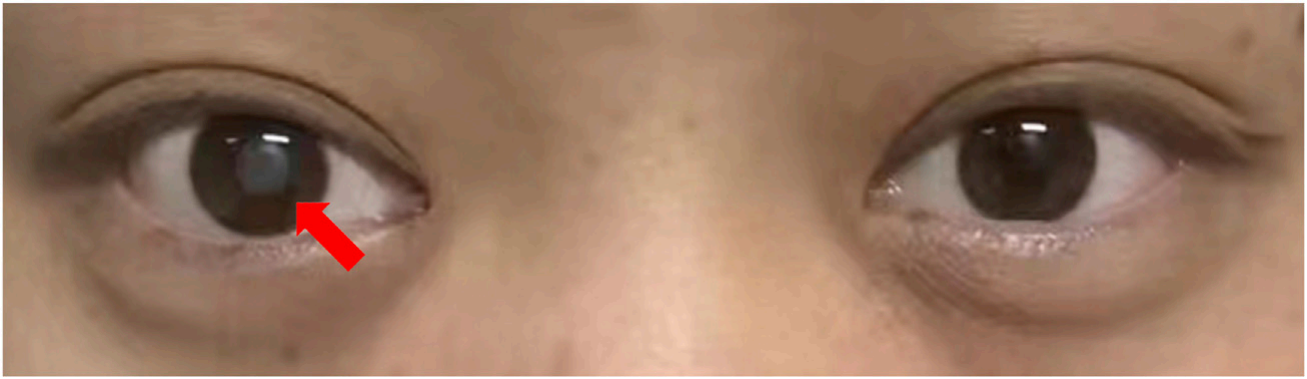
After the fifth cycle of chemotherapy for testicular cancer, the lens of the right eye was completely white and cloudy.

However, blurred vision occurred after the fifth cycle for testicular cancer treatment. He had no history of eye disease. No record of visual problems was found in his family members (Figure 2). Ophthalmic examination showed that the lens of the right eye was completely opacified, the fundus was not visible, and the and the lens of the left eye showed incomplete opacification. Visio oculus dexter for the right eye (VOD). VOD: consisted of hand motions, Visio oculus sinistra (VOS) for the left eye. VOS:0.4. Tension of the oculus dexter (TOD) was 11 mmHg and tension of the oculus sinister (TOS) was 10 mmHg. The B-ultrasound imaging of both eyes indicated opacity in the anterior crystalline region of the right eye (Supplementary Figures S3). Ultimately, cataract was diagnosed. The vision impairment in his right eye seriously affected his quality of life. To treat the cataract, the patient received phacoemulsification and replaced intraocular lenses on 19 October 2022. The postoperative ophthalmic examination showed VOD:0.4. Subsequently, his follow-up visits to the ophthalmic clinic showed VOD improved to 0.4, and patient visual acuity remained at VOD 0.4, after wearing glasses (VOD:1.0).

**FIGURE 3:**
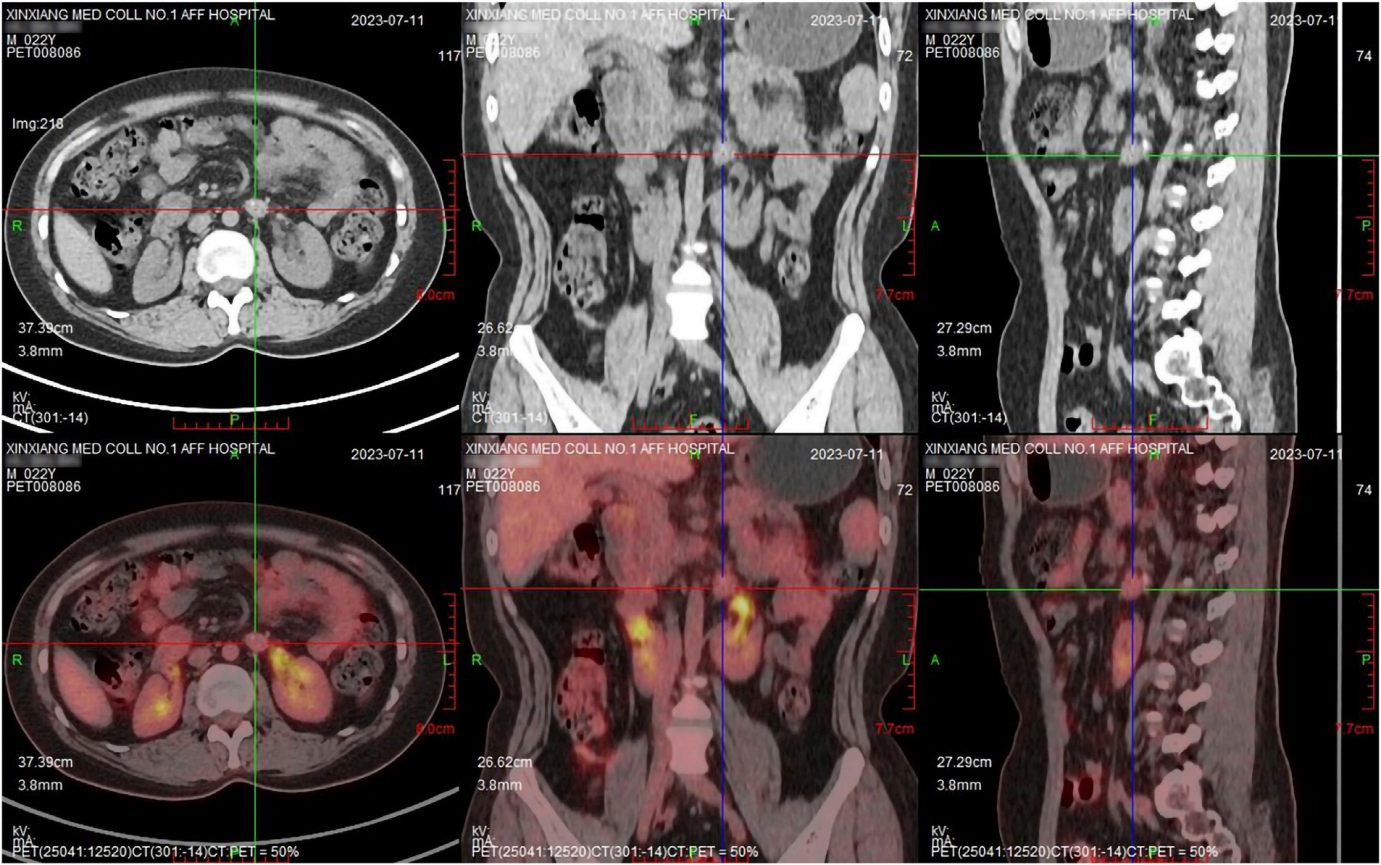
PET-CT after radiotherapy showed the metabolism in the lesion did not increase and enlarged lymph nodes were not found.

**FIGURE 4:**
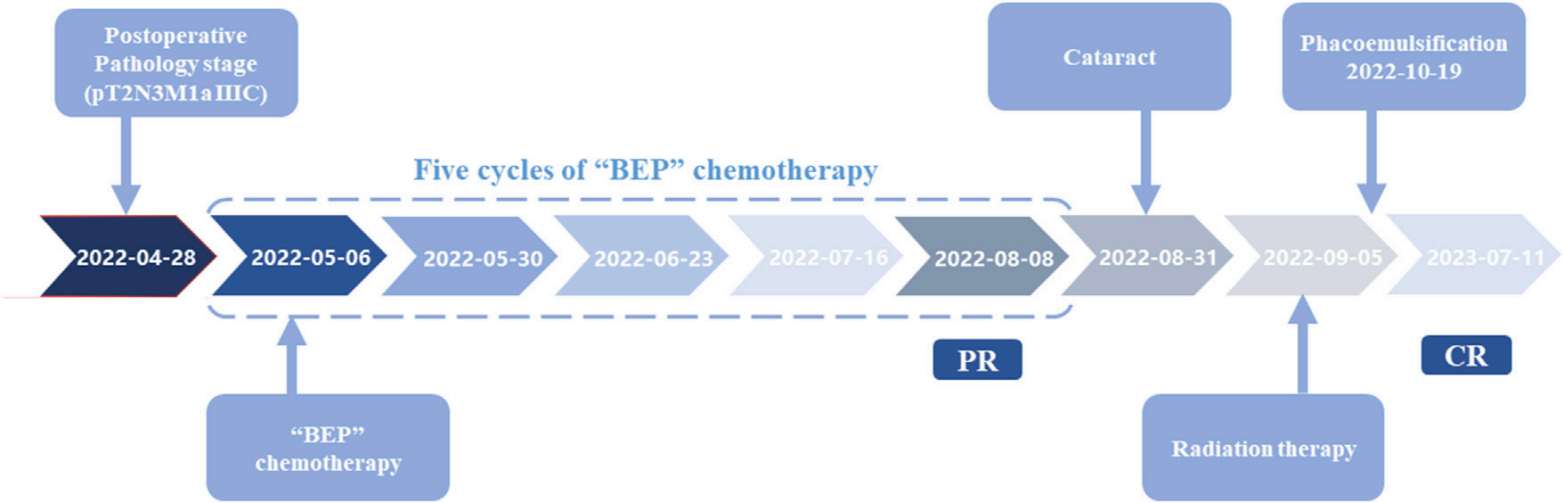
Overview of the whole treatment process.

Subsequently, antitumor therapy was modified to radiation therapy. After radiotherapy, the MRI showed the target lesion was further reduced. After 10 months, this patient underwent a PET-CT scan, which showed a nodular shadow of the left adrenal gland with calcification (15.0 mm*12.0 mm*17.0 mm) and no evidence of residual highly active tumor tissue anywhere in the body, indicating complete response (CR) (Figures 3, 4).

## 3 Discussion

The BEP regimen is a significant chemotherapy regimen with potent antitumor activity. To date, it is still the standard therapy for testicular cancer. According to a recent report, BEP remains superior to retroperitoneal lymph node dissection in preventing recurrence of non-seminomatous germ cell tumors of stage I (Hiester et al., 2021). In this case, according to the stage of cancer stage (pT2N3M1a IIIC stage), we believe that the use of the BEP regimen was the most effective antitumor strategy. However, during antitumor treatment, our patient was diagnosed with a severe cataract and finally received ophthalmic surgery.

Various factors including developmental abnormalities, trauma, metabolic disorders, and drug-induced changes may trigger the development of cataract (Ang and Afshari, 2021). Many antitumor drugs can induce ocular toxicity, and at the same time limit the progression of cancer. Fluorouracil, methotrexate can induce ocular irritation, busulfan and methotrexate can induce cataracts, mitotane, tamoxifen can induce retinopathy (Bader et al., 2023). However, etoposide and cisplatin in BEP chemotherapy, have no reports of cataract induced toxicity with their use. The etoposide is an inhibitor of topoisomerase II, which has shown antitumor activity in a variety of tumor types, including testicular cancer. Leucopenia was the major toxicity, non-hematologic toxicity was rare and included alopecia, nausea, and mucositis (Alsdorf et al., 2019). Etoposide rarely causes ocular toxicity even in high doses. Since it has been applied as an antitumor drug, there have been few reports of etoposide-related optic neuritis. Cisplatin is an antineoplastic agent, which was approved in 1978 for clinical use in the treatment of cancer. Today, it still plays a vital role in treating metastatic testicular, ovarian carcinoma, and advanced bladder cancer. As a platinum drug, the main side effect of cisplatin is neurotoxicity. The toxicity of cisplatin is usually cumulative, always induced by paresthesia, diminished or absent deep tendon reflexes, reduced sense of vibration, and decrease in position. Cisplatin can cause optic neuritis, papilledema, and even acute vision loss, usually in the fundus or retina, which can lead to blindness in severe cases (Qi et al., 2019). Related data have shown that cisplatin and etoposide can induce optic neuritis and can even induce acute vision loss. However, the lens and optic nerve are in different anatomical positions, optic neuropathy cannot cause the lens to become opaque, and the symptoms of optic neuritis are quite different from those of cataract. Throughout this therapy, the patient got supportive therapy including esomeprazole, fosaprepitant, and dexamethasone. There are no reports or studies on the adverse effects of esomeprazole and fosaprepitant on vision. Dexamethasone has been reported to cause cataracts, but this usually happens following long-term high-dose use (James, 2007). In our case, dexamethasone was used only during chemotherapy and was promptly tapered and discontinued without long-term or high-dose use. Besides that, steroid-induced cataracts usually present as posterior subcapsular cataracts with the lesion located in the posterior central position (James, 2007). In this case, the lesion was located in the anterior part of the lens.

BEP chemotherapy varies in different regions in terms of administration time and dose (Satoh et al., 2015; Wang et al., 2021). Typically, the cumulative dose of bleomycin is limited to approximately 400 mg (400,000 IU) (Chaudhary and Haldas, 2003). In our case, after 5 cycles of BEP chemotherapy, the cumulative dose of bleomycin remained below the safety threshold (195,000 IU vs. 400,000 IU). In addition, a study found that the use of 5 cycles of BEP chemotherapy increased the efficiency without a significant increase in adverse effects (Akasaka et al., 2022). According to several preclinical trial reports, Bleomycin can induce cataract in male rats (Edwards et al., 1975). As discussed above, excluding the possibility that other drugs used by the patient may cause cataracts, we surmise that bleomycin in BEP chemotherapy may be related to the development of cataracts. Bleomycin is a familiar antitumor drug. Early in 1975, exist preclinical trials reported that several newborn rats had developed cataract after receiving bleomycin treatment after 12–15 days (Edwards et al., 1975). Furthermore, in a preclinical trial published in 2022, the use of 6 days of bleomycin can induce cataract in *ex vivo* male Sprague-Dawley rats. The cortical opacity induced by bleomycin can increase in a concentration and time-dependent manner (Yamaoka et al., 2022). In our case report, based on the patient’s history and relevant drug data, we speculate that bleomycin is likely related to the development of cataracts. However, the pathogenesis of bleomycin-inducing cataracts is still incompletely understood. Cataractous-crystallins may participate in this progression (Weill et al., 1980).

## 4 Conclusion

Unlike other adverse effects induced by chemotherapy such as neutropenia, anemia, thrombocytopenia, and lung fibrosis, cataract is an extremely rare adverse event. Until now, there are still methods to effectively prevent this adverse event. When cataracts develop, cataract surgery, such as phacoemulsification and replacement of intraocular lenses, are the only way to treat cataract. The previous bleomycin-based chemotherapy scheme must be interrupted due to cataract development, which also increased the risk of progression of malignancies. Therefore, it is necessary to identify the mechanism by which bleomycin induces cataracts. By being aware of the potential side effects of bleomycin, doctors, and especially oncologists, can help patients stop taking these drugs earlier and prevent further damage. The assistance of an ophthalmologist for diagnosis and treatment should be considered if needed.

## 5 Patient perspective

Written informed consent from the patient for the use of figure and publication of their case details has been obtained by the authors.

## Data Availability

The original contributions presented in the study are included in the article/Supplementary Material, further inquiries can be directed to the corresponding authors.
