# Supplementary material for: Case report: a cataract induced by bleomycin in a patient with testicular cancer
Source: Front Pharmacol. 2024 Jun 20;15:1339662. doi: 10.3389/fphar.2024.1339662 (PMC11222336; doi:10.3389/fphar.2024.1339662)

Supplementary Material

# Figures

**Figure 1.** Comparison of abdominal magnetic resonance imaging **(a, b, c)** before and **(d, e, f)** after chemotherapy, the left retroperitoneal lymph nodes were significantly reduced.


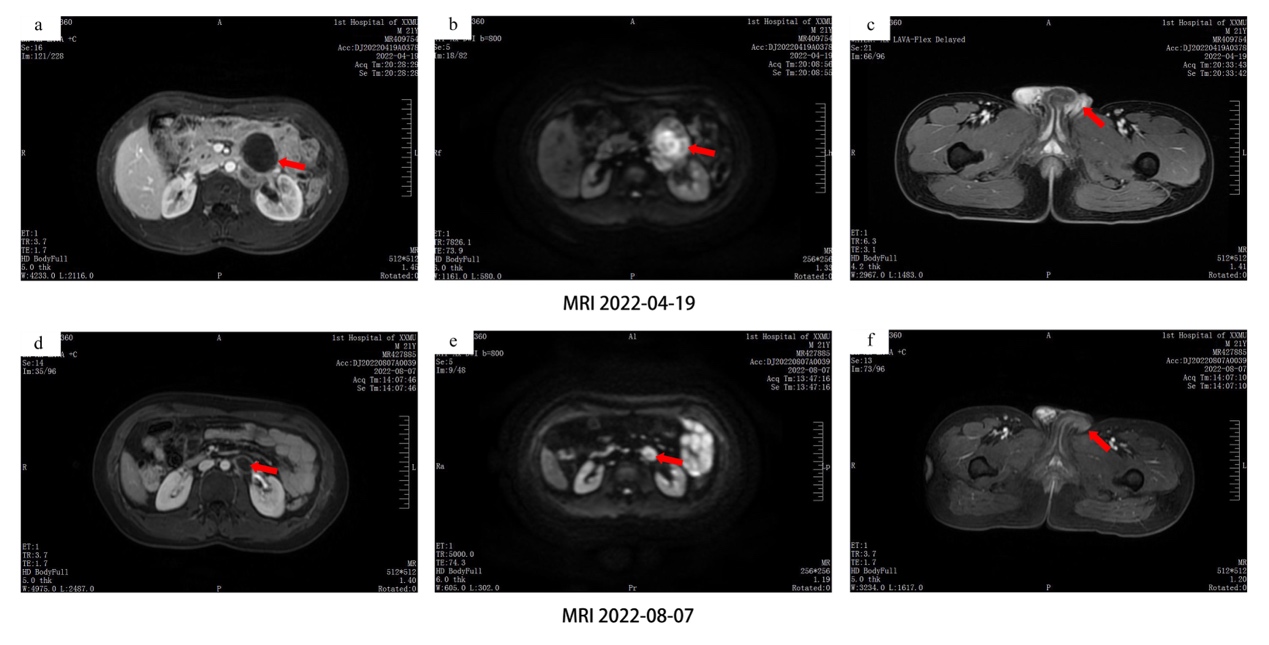


**Figure 2.** After the fifth cycle of chemotherapy for testicular cancer, the lens of the right eye was completely white and cloudy.


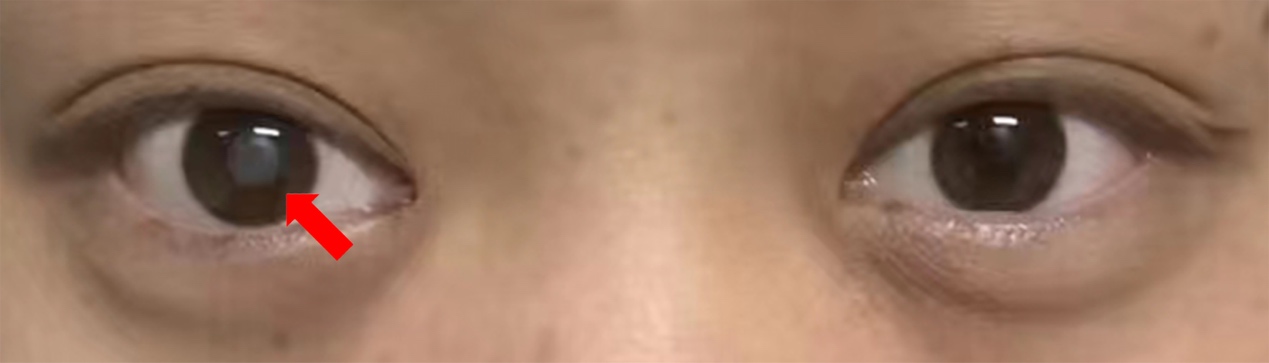


**Figure 3.** PET-CT after radiotherapy showed the metabolism in the lesion did not increase and enlarged lymph nodes were not found.


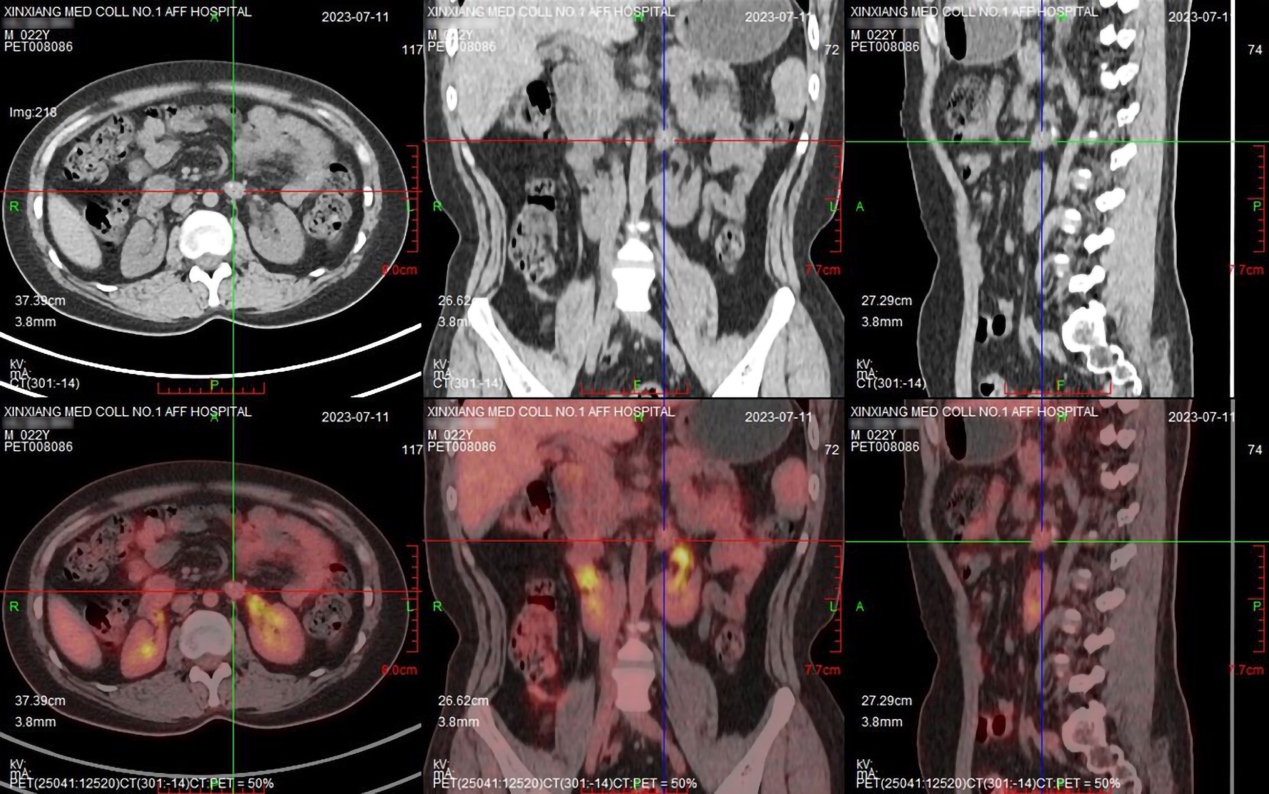


**Figure 4.** Overview of the whole treatment process.
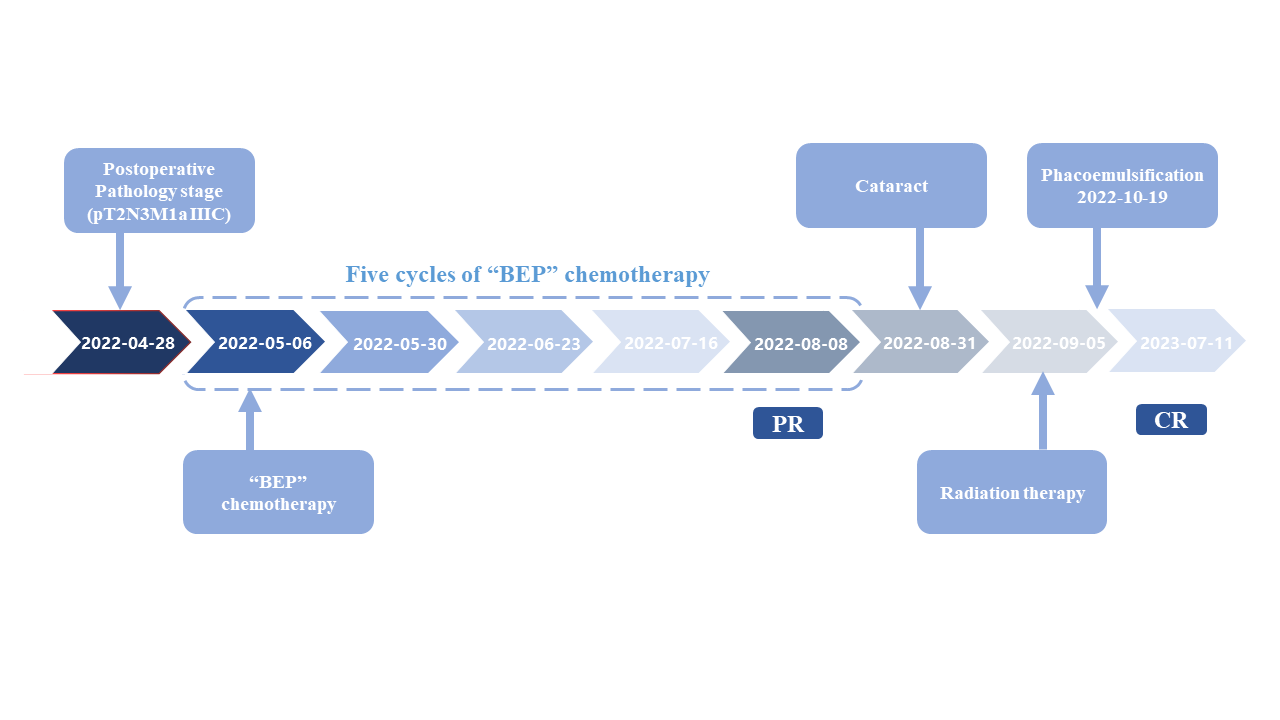


# Supplementary Figures

**Figure S1.** Histopathological features of testicular cancer. Original magnification: (a) 4×, (b) 10×, (c) 20×, (d) 40×.


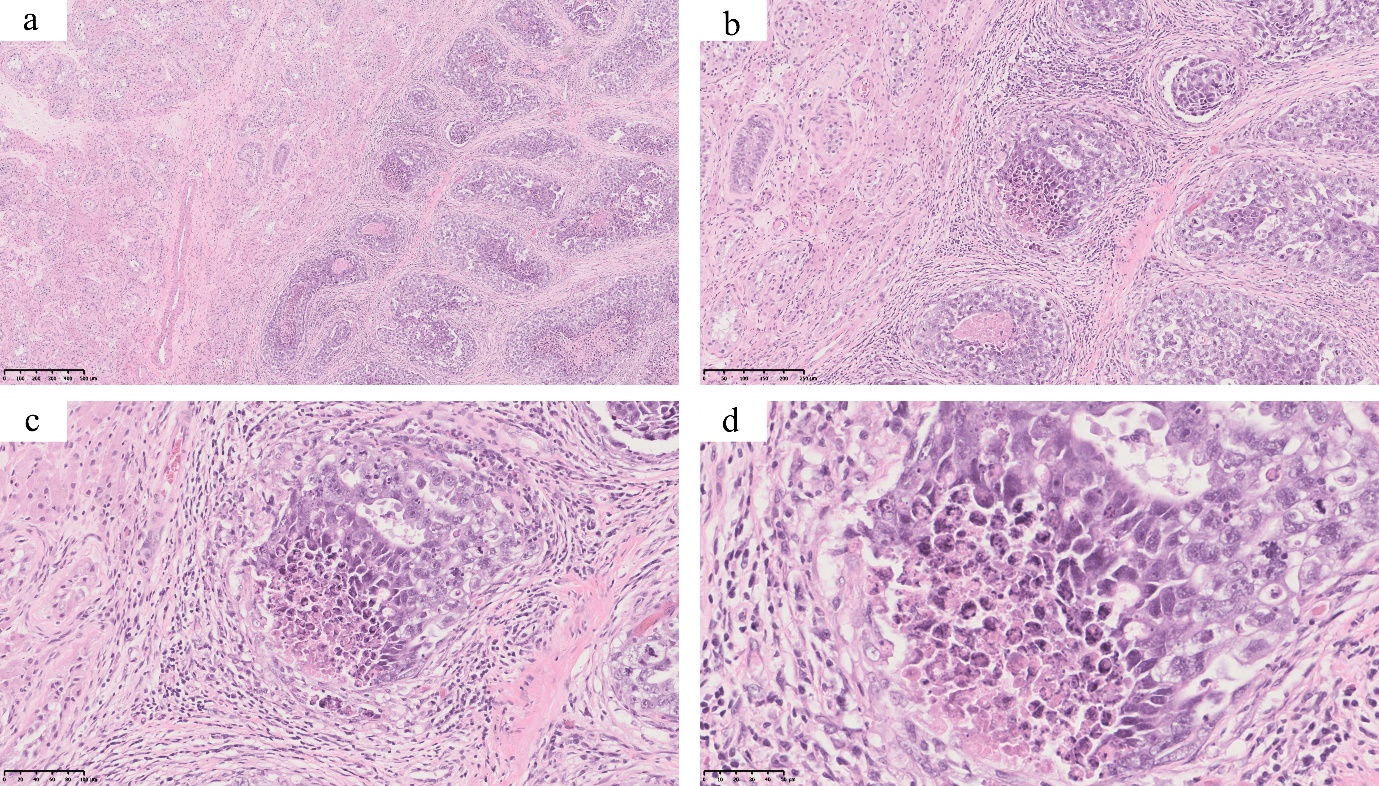


**Figure S2.** Immunohistochemistry for pathological examination. Immunohistochemistry showing malignant cells immunoreactive for CD30 and PLAP, Immunoreactive for CD30, original magnification: (a) 10×, (b) 20×. Immunoreactive for PLAP, original magnification: (c) 10×, (d) 20×.


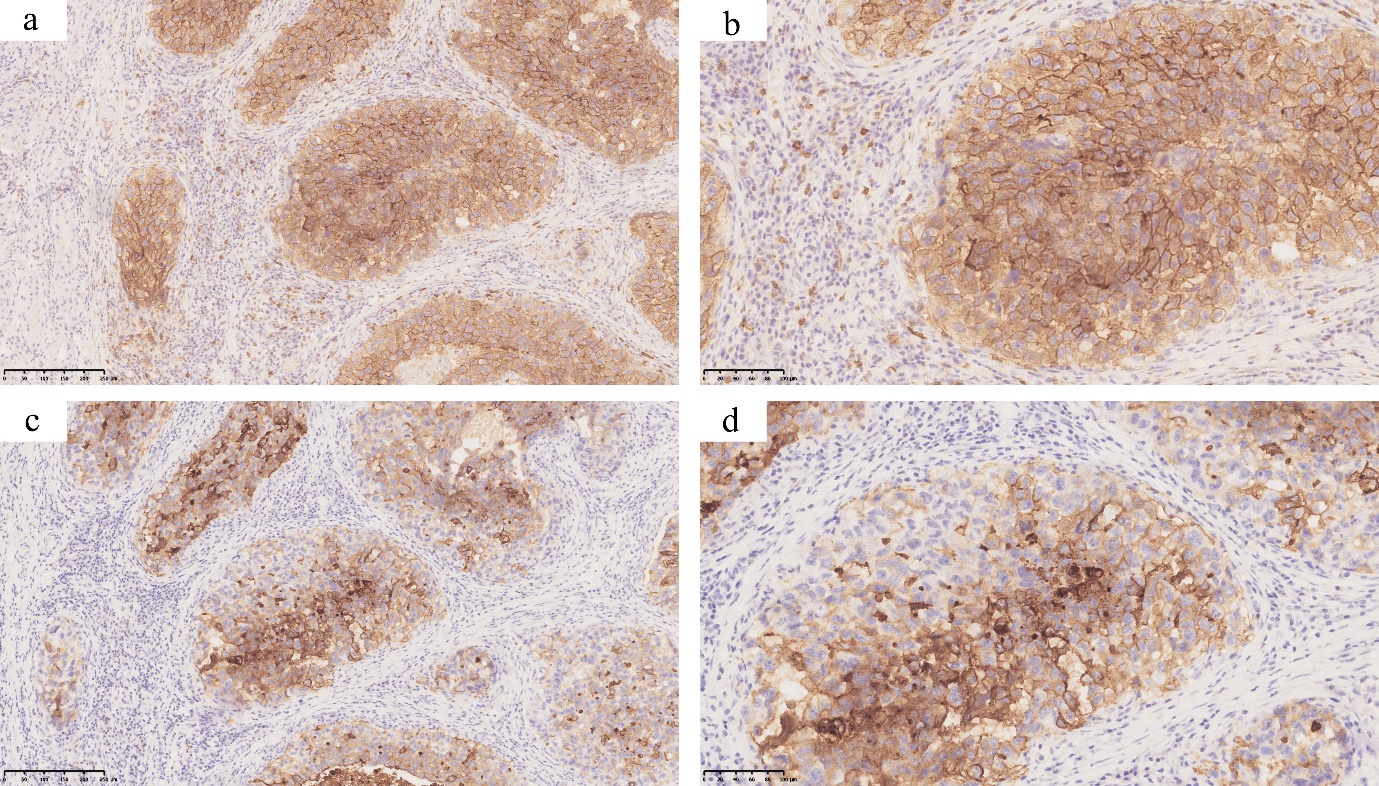


**Figure S3.** B-ultrasound imaging of both eyes indicated opacity in the lens of the right eye.


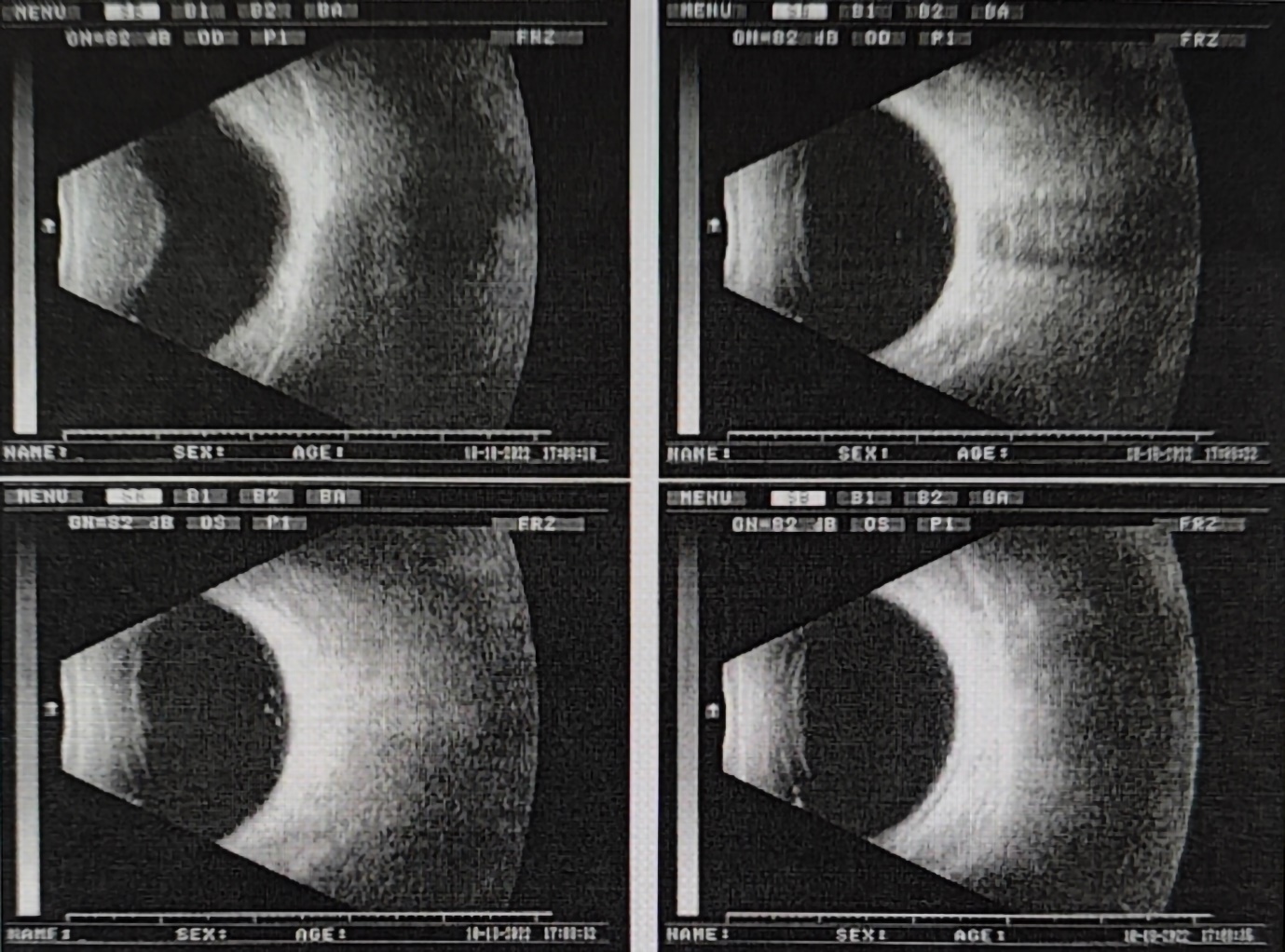

Supplement: Supplementary file 1 [file DataSheet1.docx]
